# Application of Ultra-Wide Band Sensors in Mining

**DOI:** 10.3390/s23010300

**Published:** 2022-12-28

**Authors:** Katja Wisiak, Michel Jakić, Philipp Hartlieb

**Affiliations:** Department Mineral Resources Engineering, Montanuniversität Leoben, 8700 Leoben, Austria

**Keywords:** ultra-wide band, sensors, sensor measurements, mining

## Abstract

Ultra-wideband (UWB) sensors are a radio frequency technology that use wireless communication between devices to precisely determine the position. The most recent applications focus on locating and sensor data collecting on mobile phones, car keys and other similar devices. However, this technology is still not being utilized in the mining sector. To overcome this gap, this perspective offers implementation options and solutions. Additionally, it evaluated the benefits and drawbacks of using ultra-wideband for mining. The measurements provided were made using QORVO two-way ranging sensors, and these were compared to theoretical and existing technological solutions. To ensure the optimal use of UWB sensors, a special emphasis was placed on certain influencing factors, such as ways of locating via UWB and factors affecting measurement accuracies, such as the line of sight, multipath propagation, the effect of shielding and the ideal measurement setup. A conducted experiment showed that the most accurate results are obtained when there is no multipath propagation and the arriving signal travels directly from the transmitter to the receiver.

## 1. Introduction

Ultra-wideband (UWB) is a radio technology which enables short-range, high-bandwidth communications at very low energy levels, and it covers a large portion of the radio spectrum. There are still many uses for this technology, even though it was introduced in 1901 [1]. In contrast to other similar technologies, it is not connected to any frequency. UWB can also leverage unused frequency capacity and a very wide frequency range to send data. The minimum UWB frequency range is 500 MHz [2]. In general, a signal’s frequency response and pulse width determine the accuracy that can be achieved. With UWB, the response frequency can be between 10 and 40 MHz, and the pulse width can even be as low as one nanosecond, giving it a theoretical accuracy of one centimetre [3]. The most recent applications are focused on tracking, accurate positioning, and sensor data collection. High-end smartphones and the newest cars have UWB capability that could be implemented into the mining positioning network [4]. It enables quick and reliable data transmission across small distances. UWB is widely used for indoor localisation of moving assets in complicated and space-constrained environments because of its precision, transmission speed, and dependability; this makes it ideal for usage in mining. A transmitter sends billions of pulses over the wide spectrum frequency (UWB was previously known as “pulse radio” and was most typically used in military communication applications); a corresponding receiver then converts the pulses into data by identifying a recognisable pulse sequence delivered by the transmitter. The ability to transmit pulses at a rate of one per two nanoseconds contributes to its real-time precision [3]. Depending on the requirements, one of the following four methods for position calculations can be used: time-difference of arrival (TDoA); two-way ranging (TWR); the angle of arrival (AoA); and phase-difference of arrival (PDoA) [5]. This paper presented the possibilities of using UWB technology in underground mining scenarios. Theoretical and technological solutions were compared with measurements in a real mining environment. A special focus was set on assessing the significance of the key influencing factors and constraints in such environments, i.e., limited line of sight, and cornering capacities. The conducted experiments present the advantages and possibilities of this already existing technology, and tests possible problems that might occur upon implementation in a new environment (the mining environment).

## 2. Test Setup

**Figure 1:**
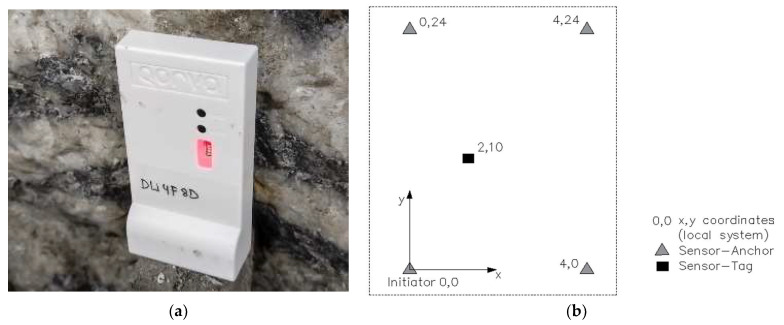
(**a**) QORVO sensor tag attached to the drift wall; and (**b**) position of the anchors and moving target.

The test was conducted using MDEK1001: Module Development and Evaluation Kit for the Decawave DWM1001C (UWB/Bluetooth module using Decawave’s DW100IC, TWR) (Figure 1a), a tablet running on an Android application (Amazon fire HD10), and CR123 batteries. The RTLS units can be powered with USB main power supplies, but for simplicity, batteries were used in this test. The moving target (Sensor tag) was simulated with a small remote-controlled car. The first step is creating a working network with built-in software. To get the measurements started, there are two options to position the anchors. The first possibility is the auto-position mode for maximum of four anchors or manually positioning the anchors. The used position in this test was auto-position mode with four anchors. The first anchor in the list with the local coordinate (0,0) must always be the initiator anchor. The next step is to measure and start the auto-positioning, in the preview modus check the locations before saving. After successful auto-positioning each device is listed in the network detail screen. The ranging starts automatically and there is an option to automatically visualize the grid and zoom on the device [6].

### Geometry Arrangement of the Sensors

The layout used for the anchor-sensor position is a rectangular shape (Figure 1b). The maximum distance reached between two sensors without signal loss in this test was 28 m. Due to the space restrictions in the test mine, the maximum width was 4 m. Furthermore, it was important that the anchors were positioned at an average height of over 1.2 m above the moving target; anchors were placed at the same vertical level. Another consideration was the roughness and irregularities of the mine wall surface on site for anchor placement. Sensors were attached to the drift wall’s existing indentations and notches, demonstrating the simplest conceivable, close-to-reality application. 

## 3. Test Results and the Factors Affecting Measurement Accuracy

### 3.1. Line of Sight (LOS)

**Figure 2:**
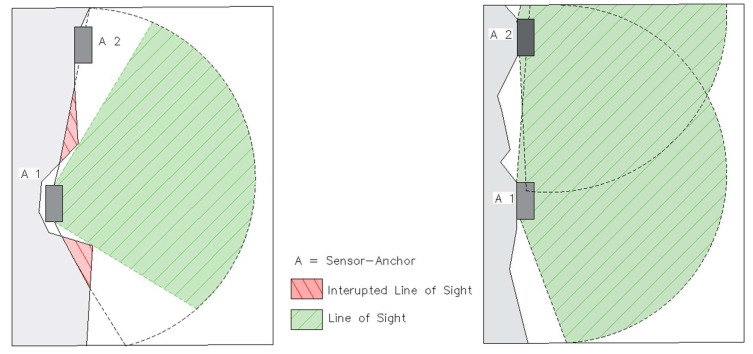
Depictured are Sensor Anchors A1 and A2 in different scenarios, on the left side is interrupted Line of Sight whereas on the right side we have clear Line of Sight from A1 to A2.

The ideal situation for the best range and accuracy is a clear line of sight. It indicates that all the anchors can ‘’see’’ one another (have a clear line of sight) [7]. A field test confirmed that an accurate result can only be achieved if all the sensors are in direct LOS. Figure 2 on the left side shows a sketch of a specific positioning of the sensor in the rough mine wall, loss of signal occurs when one of the sensors is positioned without a direct line of sight and Figure 2 on the right side shows two sensors in the line of sight (without any obstacles of the mine wall).

### 3.2. Potentially Interfering Signals

UWB is used in smartphones, along with other technologies like wireless local area network (WLAN), Bluetooth, and GPS at the same time, without any interference problems. Conducted tests also showed no interference with existing networks in underground mine environment (the local WLAN or radiocommunication system). 

### 3.3. Shielding

**Figure 3:**
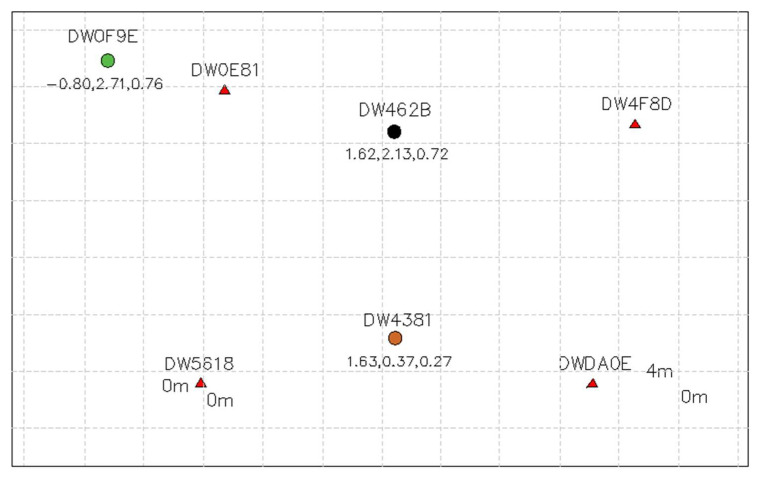
Sensor data consisting of four anchors (red triangles), 2 parallel targets (brown and black dot in between triangles) and one moving target (green dot at the top left). The numbers shown beneath the symbols are the coordinates of the sensors in 3D (x, y, z-axes) with the initiator anchor at the bottom left with (0,0,0) coordinates.

**Figure 4:**
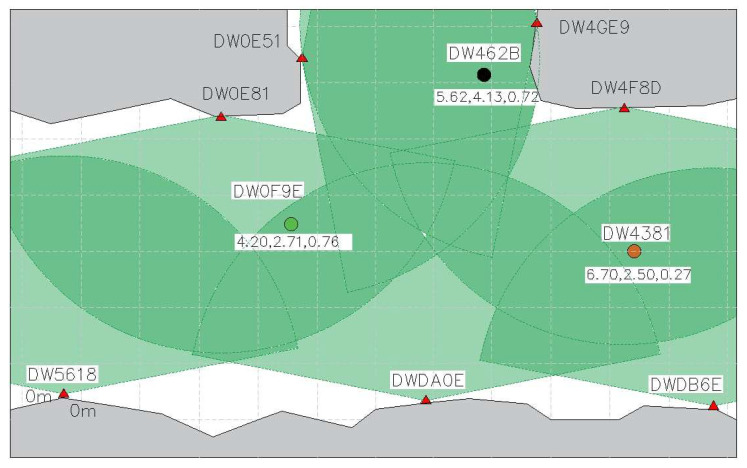
Sketch of a possible sensor layout to mitigate shielding. The light green areas are recognized by a single sensor, and the dark green areas are covered by several sensors whose effective radius are overlapping.

Tests with handheld UWB sensors and the sensor attached to the moving target demonstrated that the proposed solutions could provide accurate single-sensor localization and tracking. However, the obtained results in so-called multiple moving scenarios are not as precise as in single sensor scenarios. This can be explained by the mutual shielding (shadowing) of sensors in the observed area. When electromagnetic waves in the UWB frequency band illuminate something, a shadow zone forms behind it in the direction of the electromagnetic wave propagation. The shadow region is an area that is shielded from radar illumination by an object that reflects and/or absorbs electromagnetic waves emitted by the radar [8]. Figure 3. shows the map with sensor position in a possible shielding scenario. The red triangles are the four anchors, the brown and the black dot in between them are two fixated targets. The green dot depicts our mobile testing object. The effect of shielding was clearly evident when the mobile target was maneuvered through the two parallel sensors. As the mobile target passed between the two other targets, the one further away from the initiating sensor would disappear from the map. A choice to mitigate shielding could be a sensor configuration shown in Figure 4. Due to the overlaps, mobile tags that are in proximity to each other could more clearly be identified.

## 4. Discussion

Scientific interest in the usage of UWB is increasing every day. The automotive industry, which is just entering the age of autonomous driving, is already looking at different ways to use UWB. For example, UWB is being used to assess different collision avoidance system concepts for automobiles. Two-way ranging at 6.35 GHz achieved decimetre-level precision up to a distance of 300 m between two vehicles. Others used 2.4 GHz and 5.9 GHz radios for time difference of arrival measurements, with up to 90 m line of sight errors of less than 0.7 m depending on the velocity of the vehicles [9]. There will be many more self-driving vehicles underground or autonomous robots in the future. For that to be implemented in a safe way line of sight is a necessary component in all corners and crosscuts; strategic positioning is crucial. This is important aspect to consider for the application in the underground mining environment where, rough surfaces, and complicated geometries with many crosscuts and corners are integral parts of mining methods and layouts. If resolved successfully, it could open many new possibilities in the field of UWB navigation. The interaction of sensors on mobile devices is especially beneficial in confined spaces. The large deployment of UWB based gadgets (e.g., car keys, mobile phones) could contribute to increasing tracking capacities in any underground situation, cost-effectively including new people and machines easily into the mine safety system. For example, one may quickly identify whether someone is in a dangerous area (or close to one) and, if so, how to defuse the situation by interconnecting the sensors and heavy, moving machinery using an emergency shutdown principle in cases of need. Due to the general current uptake in UWB technology, a growing number of sales are being made in this specialized market, which will certainly result in more interesting future advances in this industry.

## 5. Conclusions

UWB sensors are currently not used in underground mining despite all the benefits and potential; the perspective offered in this paper shows that it is possible to implement this technology. The in-situ experiment demonstrated that the most precise results are obtained when there is no multipath propagation and the arriving signal travels directly from transmitter to receiver. The short UWB impulse is beneficial in line of sight and multipath propagation scenarios because it allows for the resolution of individual multipath components. Because of the short pulse duration, a UWB system can isolate the first arriving signal from later arriving reflections with the appropriate firmware. As a result, in multipath scenarios, UWB outperforms other technologies. Also, it would be beneficial to position the sensors as high as possible to have an overview of the area. To ensure continuous monitoring of moving targets, they should be positioned beneath the sensors [10]. Also, it must be taken into consideration that the quality of the sensor and device used to receive data has an impact on how precisely the positioning is done.

Instead of using only one of the above-mentioned signal parameters, hybrids of two, such as ToA/AoA or TDoA/AoA are used to obtain more accurate position information, depending on feasible processing durations, complexity constraints etc., [11].

GPS, RFID, and optical systems were used primarily for dynamic position tracking. However, GPS can only be used outdoors, RFID signals have a low accuracy of less than one meter, and optical sensors require line of sight. Other systems, such as Wi-Fi and Bluetooth, consume significantly more power than UWB. External factors have little impact on UWB. UWB technology can act as an excellent and cost-efficient means for tracking and tracing people and machines in underground situations. This ultimately makes it valuable as a supplementary technique in existing safety and monitoring measures. In order to have more data and precise results, more tests are necessary. It would also be beneficial to include other available sensors from various producers to compare their precision and correlate them with more parameters.

The conducted tests results showed that:

- the maximum distance between two sensors without signal loss between them in this test was 28 m;
- sensor tags must be positioned above the moving target at least 0.5 m in order to gain best precision;
- more moving targets at once can cause shielding and possible loss of signal for one moving target in a specific positioning, and to mitigate this more sensors are necessary;
- other devices and signals (wireless local area network (WLAN), Bluetooth, radiocommunication system and GPS) do not cause any interference with UWB sensors.

## References

[1] Nekoogar F. (2006). Ultra-Wideband Communications: Fundamentals and Applications/Faranak Nekoogar.

[2] Zhou Y., Law C.L., Xia J. Ultra low-power UWB-RFID system for precise location-aware applications. Proceedings of the 2012 IEEE Wireless Communications and Networking Conference Workshops.

[3] Zhang K., Shen C., Gao Q., Wang H. Research on similarity metric distance algorithm for indoor and outdoor firefighting personnel precision wireless location system based on vague set on UWB. Proceedings of the 2017 IEEE 17th International Conference on Communication Technology (ICCT).

[4] Inpixon Ultra-Wideband (UWB) Positioning & Sensor Technology. https://www.inpixon.com/technology/standards/ultra-wideband.

[5] UWB in Detail: Technical Specifications. https://kinexon.com/uwb-technology/.

[6] Qorvo I. MDEK1001 Ultra-Wideband (UWB). https://www.qorvo.com/products/p/https//www.qorvo.com/products/p/MDEK1001.

[7] Haslett C. (2008). Essentials of Radio Wave Propagation.

[8] Kocur D., Fortes J., Švecová M. (2017). Multiple moving person tracking by UWB sensors: The effect of mutual shielding persons and methods reducing its impacts. J. Wirel. Commun. Netw..

[9] Petovello M.G., O’Keefe K., Chanv B., Spiller S., Pedrosa C., Xie P., Basnayake C. (2012). Demonstration of Inter-Vehicle UWB Ranging to Augment DGPS for Improved Relative Positioning. JGPS.

[10] Jimenez A.R., Seco F. Comparing Decawave and Bespoon UWB location systems: Indoor/outdoor performance analysis. Proceedings of the 2016 International Conference on Indoor Positioning and Indoor Navigation (IPIN).

[11] Elbahhar F., Fall B., Rivenq A., Heddebaut M., Elassali R., Elbahhar F. (2012). Indoor Positioning System Based on the Ultra Wide Band for Transport Applications. New Approach of Indoor and Outdoor Localization Systems.

